# Mechanism of rhubarb in the treatment of hyperlipidemia: A recent review

**DOI:** 10.1515/med-2023-0812

**Published:** 2023-10-03

**Authors:** Lijiao Wu, Xiangjin Wang, Jihang Jiang, Yong Chen, Bo Peng, Wei Jin

**Affiliations:** Chengdu University of Traditional Chinese Medicine School of Clinical Medicine, Hospital of Chengdu University of Traditional Chinese Medicine, Chengdu, China; College of Sports Medicine and Health, Chengdu Sports University, Chengdu, China; Emergency Department, Hospital of Chengdu University of Traditional Chinese Medicine, Chengdu, China; Respiratory Department, Hospital of Chengdu University of Traditional Chinese Medicine, Chengdu, China

**Keywords:** rhubarb, lipid metabolism, Chinese herbal medicine, hyperlipidemia, mechanism, lipid lowering

## Abstract

Hyperlipidemia is a metabolic disorder, which is a major risk factor for atherosclerosis, stroke, and coronary heart disease. Although lipid-lowering treatments have been extensively studied, safer treatments with fewer adverse effects are needed. Rhubarb is a traditional Chinese medicine that has lipid-lowering, anti-inflammatory, and antioxidant properties. Disturbance in lipid metabolism is the basis of tissue damage caused by hyperlipidemia and plays a key role in the development of hyperlipidemia; however, the molecular mechanisms by which rhubarb regulates lipid metabolism to lower lipid levels are yet to be elucidated. We conducted this study to summarize the phytochemical constituents of *Rheum officinale* and provide a comprehensive review of the molecular mechanisms underlying the regulation of lipid metabolism during hyperlipidemia treatment. It was found that rhubarb extracts, including emodin, rhubarb acid, and rhubarb phenol, regulate total cholesterol, triglyceride, TNF-α, and IL-1β levels through signaling pathways such as C/EBP α, 3T3-L1, PPAR α, and AMPK, thereby improving the hyperlipidemic state. This suggests that rhubarb is a natural drug with lipid-lowering potential, and an in-depth exploration of its lipid-lowering mechanism can provide new ideas for the prevention and treatment of hyperlipidemia.

## 1 Introduction

Hyperlipidemia is a disease characterized by abnormal lipid metabolism, manifested by elevated total cholesterol (TC), triglyceride (TG), and low-density lipoprotein cholesterol (LDL-C) levels and/or decreased high-density lipoprotein cholesterol (HDL-C) levels [1]. A hypercholesterolemic diet and genetic and environmental factors are important causes of hyperlipidemia [2,3]. The main pathogenesis is an increase in lipoprotein synthesis and a decrease in lipid clearance pathways, leading to abnormally elevated levels of lipids or lipoproteins in the blood, which are mainly involved in inflammatory response and oxidative stress [4,5]. Hyperlipidemia has a wide range of effects on organisms. Abnormalities in lipid metabolism induce oxidative stress and mitochondrial dysfunction, triggering structural and functional changes in the heart such as myocardial hypertrophy, apoptosis or necrosis of cardiomyocytes, atherosclerosis, heart failure, and sudden death [6,7]. Cardiovascular disease (CVD) kills approximately 17.9 million people each year globally. The risk of CVD in patients with hyperlipidemia is approximately twice as high as that in patients without hyperlipidemia [8]. Additionally, hyperlipidemia is associated with several chronic diseases, such as hypertension, fatty liver, cirrhosis, peripheral vascular disease, ischemic cerebrovascular disease, and pancreatitis [9,10]. The incidence of hyperlipidemia has sharply risen in recent years as lifestyle and eating habits have changed significantly [11]. Therefore, the prevention and treatment of hyperlipidemia to reduce the incidence of chronic diseases, such as CVDs, has become an increasing concern for society.

Currently, fibrates, statins, bile acid sequestrants, niacins, and cholesterol absorption inhibitors are commonly used to treat hyperlipidemia [12]. Although these drugs have some therapeutic effects, they cause toxic side effects, such as mild-to-moderate elevation of liver transaminases, nerve damage, myopathy, rhabdomyolysis, and increased risk of diabetes mellitus, after long-term treatment [13,14]. Therefore, it is essential to explore new therapeutic agents, and there is a growing tendency to use natural medicines to treat and prevent diseases [15,16]. A variety of plant-derived substances have excellent lipid-lowering effects, and their beneficial properties include inhibition of pancreatic lipase, reduction of dietary fat absorption, stimulation of lipolysis, and reduction of lipogenesis [17,18].

Rhubarb is a famous traditional Chinese medicine belonging to the genus Rhubarb of the Polygonaceae family. Its application can be traced back to the Shennong’s Classic of Materia Medica (270 BC) [19]. For more than 2,000 years, rhubarb has been cultivated worldwide for the treatment of constipation, diabetic nephropathy, chronic renal failure, acute pancreatitis, and gastrointestinal bleeding [20]. Recent studies have shown that rhubarb has hypolipidemic, antibacterial, anti-inflammatory, and antioxidant activities [21], and it is gradually being applied in the prevention and treatment of hyperlipidemia.

Seven databases, PubMed, SciFinder, Scopus, Web of Science, CNKI, Wipu, and Wanfang, were searched from creation of the database to November 25, 2022. We searched original studies, reviews, and newsletters in English and Chinese for search terms such as “rhubarb,” “hyperlipidemia,” “lipid metabolism,” “pharmacology,” “compounds,” “pharmacology,” “biological activity,” “clinical application,” and “toxicity.” If the literature lacked data or the report was unclear, we corresponded with the authors. If the original data remained unavailable, the literature was excluded. The bibliographies of all selected articles were also scanned for additional relevant articles, and the PubChem database was used to check the IUPAC names of known rhubarb.

## 2 Phytochemistry

Research on the chemical composition of rhubarb began in the early nineteenth century and approximately 200 chemical components [20], including anthraquinones, anthrone, stilbenes, tannins, acyl glucosides, and other bioactive compounds, have been isolated and identified. Among these components, anthraquinones, including emodin, rhubarb acid, rhubarb phenols, and their derivatives, are dominant [22,23], in addition to stilbenes containing mainly resveratrol and its derivatives. Table 1 shows the composition of 48 common compounds in rhubarb.

**Table 1:** Common chemical constituents of rhubarb

Class	S.N.	Compounds	References
Anthraquinones	1	Emodin	Verma et al. [24]
	2	Aloe-emodin	Agarwal et al. [25]
	3	Emodin-*O*-d-glucoside	Ye et al. [26]
	4	Emodin-8-*O*-β-d-glucopyranoside	Verma et al. [24]
	5	Emodin 8-*O*-β-d-glucopyranosyl-6-*O*-sulfate	Krenn et al. [27]
	6	Emodin 8-*O*-(6′-*O*-malonyl)-glucoside	Ye et al. [26]
	7	Emodin 8-*O*-(2′,3′,4′,6′-tetra acetyl)-glucoside	Krenn et al. [27]
	8	Chrysophanol	Agarwal et al. [25]
	9	Chrysophanol 1-*O*-glucoside	Ye et al. [26]
	10	Chrysophanol 8-*O*-(6′-*O*-galloyl)-glucoside	Ye et al. [26]
	11	Chrysophanol-8-*O*-β-d-glucopyranoside	Suresh Babu et al. [28]
	12	Physcion	Agarwal et al. [25]
	13	Physcion-1-*O*-β-d-glucopyranoside	Wang et al. [29]
	14	Physcion-8-*O*-β-d-glucopyranoside	Wang et al. [29]
	15	6-Methyl-aloe-emodin	Singh et al. [30]
	16	6-Methyl-aloe-emodin-triacetate	Singh et al. [30]
	17	6-Methyl-rhein	Singh et al. [30]
	18	6-Methyl-rhein-diacetate	Singh et al. [30]
	19	Rhein	Singh et al. [25]
	20	Rhein 1-*O*-glucoside	Ye et al. [26]
	21	Rhein 8-*O*-glucoside	Ye et al. [26]
	22	Torachrysone-8-*O*-β-d-glucopyranoside	Suresh Babu et al. [28]
	23	8-*O*-β-d-(6′-*O*-acetyl) glucopyranosyl-chrysophanol	Krenn et al. [31]
Anthrones	24	10-Hydroxycascaroside D	Krenn et al. [31]
	25	Anthrone C-glucosides	Krenn et al. [31]
	26	10R-chrysaloin 1-*O*-β-d-glucopyranoside	Krenn et al. [31]
	27	10-Hydroxycascaroside C or anthrone C-glucosides	Krenn et al. [31]
	28	Cascaroside C	Krenn et al. [31]
	29	Cascaroside D	Krenn et al. [31]
	30	Cassialoin	Krenn et al. [31]
Stilbenes	31	Resveratrol	Rokaya et al. [32]
	32	Resveratrol 3-*O*-β-d-glucopyranoside	Ngoc et al. [33]
	33	Resveratrol-4′-*O*-β-d-glucopyranoside	Chen et al. [34]
	34	Resveratrol-4′-*O*-β-d-(6″-*O*-galloyl)-glucoside	Chen et al. [34]
	35	Resveratrol-4′-*O*-β-d-(2″-*O*-galloyl)-glucoside	Chen et al. [34]
	36	Piceatannol	Liu et al. [35], Wang et al. [29]
	37	Piceatannol-3′-*O*-β-d-glucopyranoside	Wang et al. [29]
	38	Piceatannol-4′-*O*-β-d-(6″-*O*-galloyl)-glucopyranoside	Liu et al. [35]
	39	Piceatannol-4′-*O*--d-glucopyranoside	Liu et al. [35], Wang et al. [29]
	40	Desoxyrhaponticin	Suresh Babu et al. [28]
	41	Desoxyrhapontigenin	Suresh Babu et al. [28]
	42	Rhaponticin	Chen et al. [36]
	43	Rhapontigenin	Zhang et al. [37]
Tannins	44	d-Catechin	Krenn et al. [27]
	45	Epicatechin	Krenn et al. [27]
Phenylbutanone	46	4-(4′-Hydroxyphenyl)-2-butanone-4′-*O*-β-d-glucopyranoside	Kashiwada et al. [38]
	47	4-(4′-Hydroxyphenyl)-2-butanone-4′-*O*-β-d-(2″,6″-*O*-cinnamoyl)-glucopyranoside	Kashiwada et al. [38]
	48	Isolindleyin	Nonaka et al. [39]

**Figure 1:**
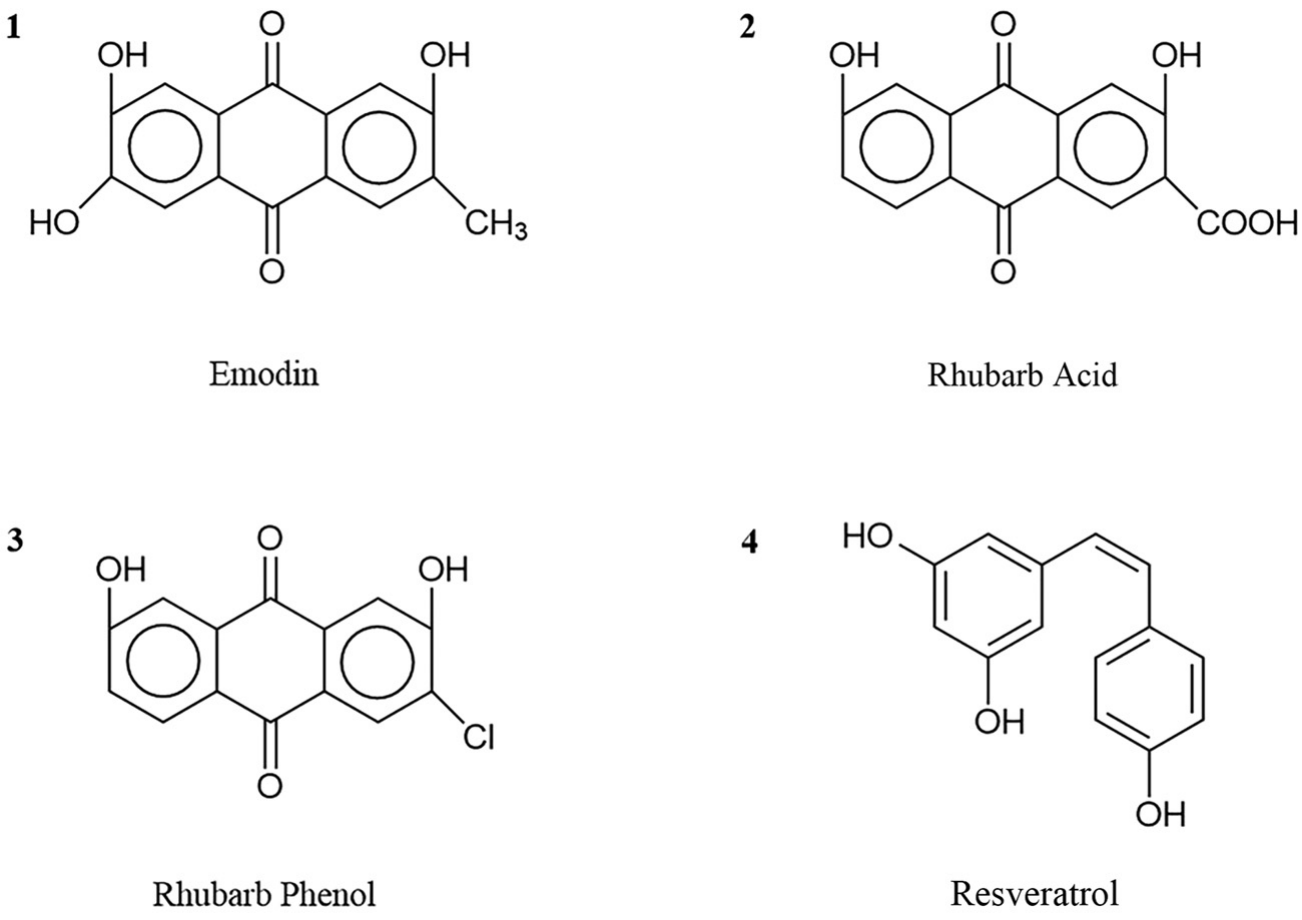
Chemical structure of the main components of rhubarb.

Anthraquinones are the predominant substances isolated from rhubarb and their most potent active component is emodin, which consists mainly of a 1,8-dihydroxy-9,10-anthraquinone skeleton. If different functional groups are attached to different parts of the backbone structure, they can display different chemical structures (Figure 1), thereby exhibiting different chemical properties and pharmacological effects [40]. For example, two chemical components, rhubarb phenols (1,8-dihydroxy-3-methylanthraquinone) and emodin (1,3,8-trihydroxy-6-methylanthraquinone), have a basic skeleton of 1,8-hydroxy-methylanthraquinone, but their functional groups are in different locations, which leads to differences in their pharmacological effects. Although both have a lowering effect on plasma lipid levels, emodin has stronger antitumor and anti-inflammatory effects and is more influential [41]. Regarding the structure–effect relationship of toxicity, 30 μM emodin induced significant apoptosis in a time-dependent manner, according to the morphological changes in L-02 cells. Additionally, rhodopsin may interfere with the metabolism of glutathione (GSH) and fatty acids in human hepatocytes [42].

## 3 Molecular mechanism of lipid metabolism regulation by rhubarb

Rhubarb is a classical laxative drug, and its pharmacological studies have shown that it can regulate lipid metabolism and has anti-inflammatory effects. Therefore, in addition to constipation, it is also used to treat disorders of lipid metabolism and hyperlipidemia. Emodin, rhubarb acid, rhubarb phenol, and resveratrol are the main substances that regulate lipid metabolism and can inhibit pancreatic lipase, reduce lipogenesis, stimulate lipolysis, and regulate lipid factor expression to achieve lipid lowering.

### 3.1 Emodin

Emodin is the predominant anthraquinone in rhubarb, and it is recognized as a protein complex kinase inhibitor with activity against a variety of tumor cells, in addition to its antioxidant, lipid metabolism regulating, and antibacterial effects.

Emodin has diverse regulatory mechanisms on lipid metabolism. It was found that rhodopsin is closely related to peroxisome proliferator-activated receptor (PPAR) γ nuclear receptor, and rhodopsin can act as its activator to regulate lipid metabolism, promoting cholesterol efflux from THP1 macrophages, up-regulating scavenger receptor BI, facilitating reverse cholesterol transport, and inhibiting cholesteryl ester accumulation by activating the PPAR γ signaling pathway [43–45]. Additionally, emodin acts directly on transcription factors to regulate lipid metabolism. Li et al. found that emodin significantly inhibited the mRNA expression of SREBP-2, a major transcription factor of cholesterol biosynthesis, and reduced the mRNA levels of cholesterol metabolism-related genes HMGCR, SS, LSS, and Sc4mol whereas increased the lipolytic mRNA levels of high-density lipoprotein receptor (SRBI), hepatic lipase, and apolipoprotein E (Apo E), showing an overall reduction in lipid synthesis and enhanced fatty acid oxidation (FAO) [46]. Xue et al. found that emodin has a regulatory effect on LPL and FAT/CD36 mRNA expression and helps improve dyslipidemia. Inflammatory factors can induce lipolysis, and emodin has a clear modulatory effect on inflammatory factors [47]. Zhang et al. found that emodin promotes lipid metabolism by down-regulating TNF-α, thereby inhibiting TNF-α-induced lipolysis [48].

Cholesterol is a precursor of bile acids, which are steroids synthesized from cholesterol in the liver [49], and the conversion of cholesterol to bile acids and their secretion into bile is one of the important ways in which the body removes cholesterol [50]. Wang et al. [51] found that the combination of rhodopsin with bile acids could reduce bile acid levels, thus, promoting the conversion of cholesterol to bile acids and contributing to the reduction of serum cholesterol. Notably, among the various mechanisms underlying the lipid-lowering effects of emodin, it inhibits both 3T3-L1 adipocytes and induces lipolysis [48]. Furthermore, emodin has concentration-dependent effects on 3T3-L1 adipocytes, and it promotes the proliferation of 3T3-L1 preadipocytes at low concentrations and inhibits their proliferation at higher concentrations [52]. Meng et al. suggested that emodin may inhibit the uptake of NPC1L1 cholesterol by human hepatocytes in an anti-competitive manner with cholesterol-lowering potential [53].

**Figure 2:**
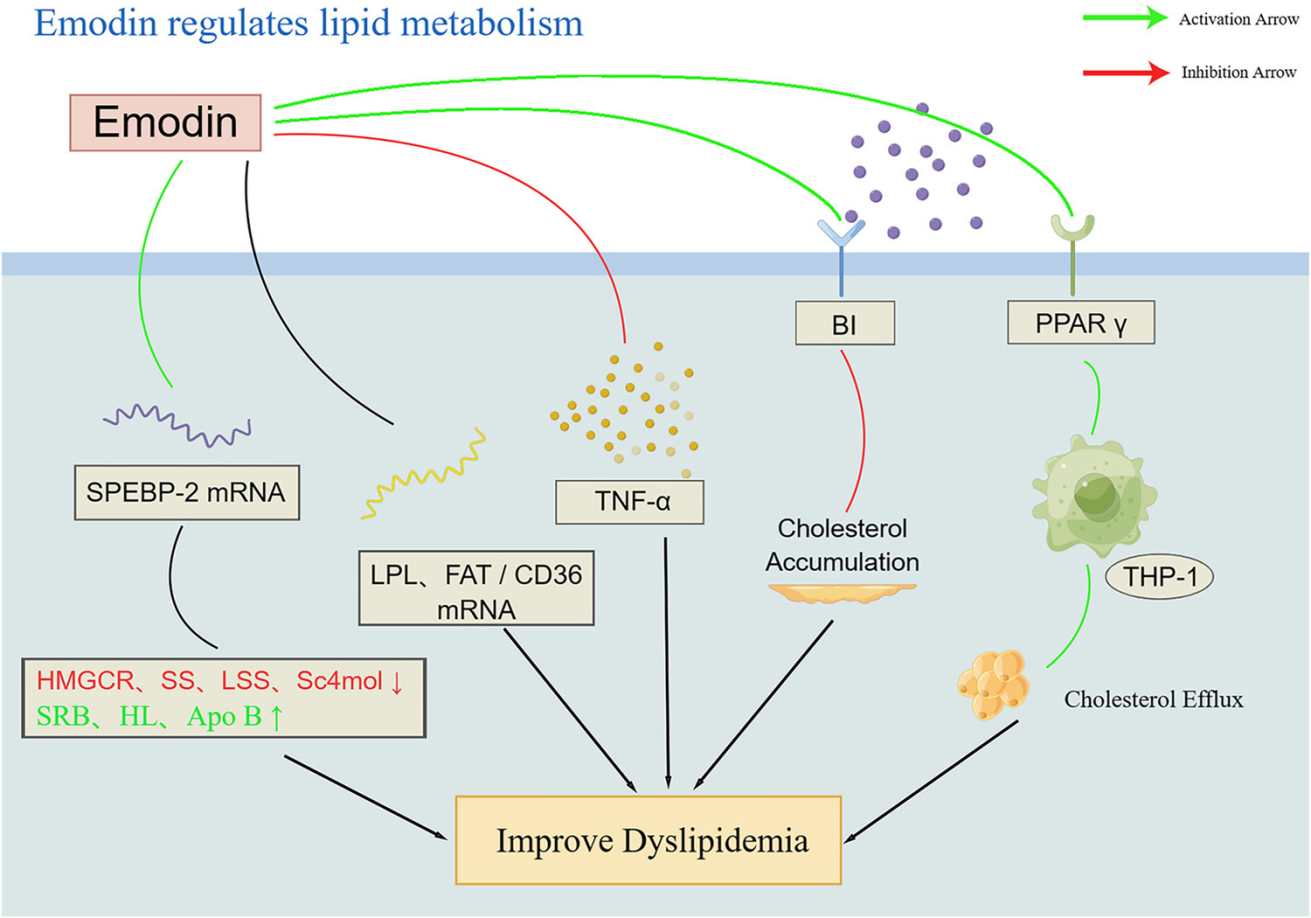
Diagram of the lipid-lowering mechanism of emodin.

The above studies have shown that the specific mechanism of action of rhodopsin in lipid-lowering mainly includes activation of PPAR γ signaling pathway, regulation of mRNA expression of lipid metabolism-related factors such as SREBP-2, SRBI, Sc4mol, etc., as well as inhibition of metabolism in 3T3-L1 adipocytes (Figure 2). Meanwhile, emodin has a scavenging effect on cholesterol, and lowering blood cholesterol in hyperlipidemic patients may have a hepatoprotective effect by improving the severity of fatty liver disease. Therefore, the relationship between emodin level and liver function requires further investigation.

### 3.2 Rhubarb acid

Rhubarb acid (4,5-dihydroxyanthraquinone-2-carboxylic acid), with a molecular weight of 284.22, is the most important active component of anthraquinones. It has a variety of pharmacological activities, such as antitumor, anti-inflammatory, anti-fibrotic, and regulation of glucolipid metabolism [54,55]. The hypolipidemic effects of rhubarb acid are of great interest. It inhibits adipocyte differentiation and significantly improves abnormal lipid metabolism in HTG [56]. Fang et al. found that rhubarb anthraquinones inhibit lipid accumulation before and after 3T3-L1 cell differentiation in 3T3-L1 adipocytes and high-fat diet (HFD)-induced obese rats, with rhubarb acid showing stronger inhibition and higher hypolipidemic activity. These effects may be related to the inhibition of PPAR γ and expression of C/EBP α transcription factors by rhubarb acid to block the production of fatty acid synthase (FAS) and acetyl-CoA carboxylase (ACC) [57]. Rhubarb acid may also lower the lipid levels by enhancing lipolysis in adipocytes. Lipolysis is a catabolic reaction in which stored TG are hydrolyzed to release glycerol and free fatty acids. The production/metabolism balance of fat cells is a prerequisite for the regulation of energy balance in body [58]. Rhubarb acid treatment increases the expression of lipolytic enzymes ATGL and HSL, which hydrolyze TG to glycerol and increase lipolysis by downregulating key lipogenic transcription factors in adipocytes [57,59]. The MAPK pathway is closely associated with adipocyte differentiation, and MAPK activation is accompanied by C/EBP β and C/EBP δ expression, which further activates PPAR γ and C/EBP α expression to oversee terminal adipocyte differentiation [60]. Rhubarb acid blocks MAPK signaling in macrophages, thereby inhibiting the transcription of pro-inflammatory mediators TNF-α and IL-1β [61,62].

**Figure 3:**
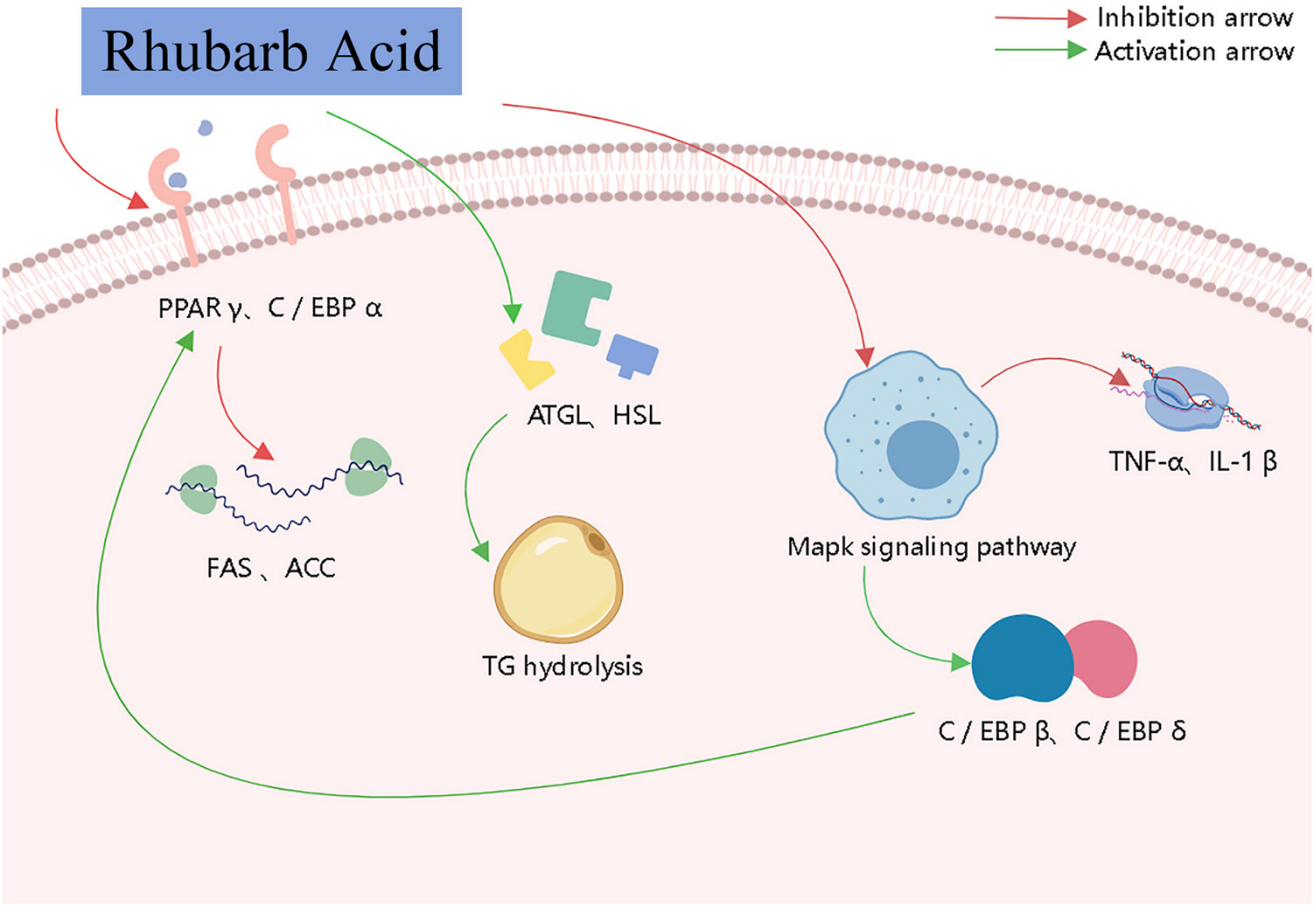
Diagram of the lipid-lowering mechanism of rhubarb acid.

Taken together, the lipid-lowering effects of rhubarbic acid are mainly mediated by inhibiting 3T3-L1 adipocytes, PPAR γ and C/EBP α transcription factor expression, and MAPK signaling, and promoting the expression of lipolytic enzymes ATGL and HSL (Figure 3). Rhubarbic acid is commonly used for lipid-lowering, weight loss, laxatives, detoxification, cleansing the internal environment, preventing gastric cancer, and delaying aging. Compared to traditional lipid-lowering chemicals, rhubarb acid is less toxic and has a hepatoprotective effect [63]. Rhubarb acid is the only anthraquinone that can be absorbed into the blood after oral administration of rhubarb extract in humans. However, it is difficult to solubilize rhubarb acid in water, and increasing its water solubility and improving the rate of drug dissolution is the breakthrough point for improving the lipid-lowering effect of rhubarb acid.

### 3.3 Rhubarb phenol

Rhubarb phenols belong to the anthraquinone group and have pharmacological effects such as neuroprotective, anticancer, antibacterial, antiviral, antioxidant, and lipid-regulating effects [64]. Studies have shown that rhubarb phenols can significantly lower blood lipid levels and reduce lipid accumulation in animals fed with HFD [65,66]. Zhang et al. found that rhubarb phenol significantly reduced the expression of FAS and ACC and increased the levels of ACOX1 and CPT1 in obese mice, thus, promoting lipolysis at the cellular and molecular levels [66]. Kwon et al. [67] found that rhubarb phenol similarly reduced lipid accumulation and expression of the lipogenic factors PPAR γ and CCAAT/C/EBP α in 3T3-L1 adipocytes. Meanwhile, Feldman et al. showed that rhubarb phenols significantly up-regulated the r RNA levels of MGLL and HSL, which are key enzymes in lipolysis, and also the expression of β-oxidation-related genes in fatty acids [68]. Liu et al. found that rhubarb phenol increased FAO in 3T3-L1 adipocytes (PPARα, Acadvl, Acadl, Acadm, L1) by exploring the effect of rhubarb phenol on lipid metabolism in obese mice. Expression of FAO (PPARα, Acadvl, Acadl, Acadm, Cpt2), lipolysis (HSL, MGLL), and thermogenic genes (Ppargc-1α, Prdm16) in L1 adipocytes suggests that rhubarb phenol promotes lipolysis, inhibits lipogenesis, and thus, inhibits lipid accumulation [69]. In terms of signaling pathways, AMPK, an AMP-dependent protein kinase, is a cellular energy receptor that promotes fatty acid metabolism and mitochondrial biosynthesis [70]. Liu et al. indicated that rhubarb phenol promotes lipolysis and reduces body weight and fat accumulation in HFD-induced obese mice by activating the AMPK pathway [69]. Li et al. found that the intensity of the hypolipidemic effect of rhubarb phenol may correlate with its concentration. Rhubarb phenol dose-dependently inhibits human SRE promoter activity and reduces intracellular cholesterol and TG levels [71].

**Figure 4:**
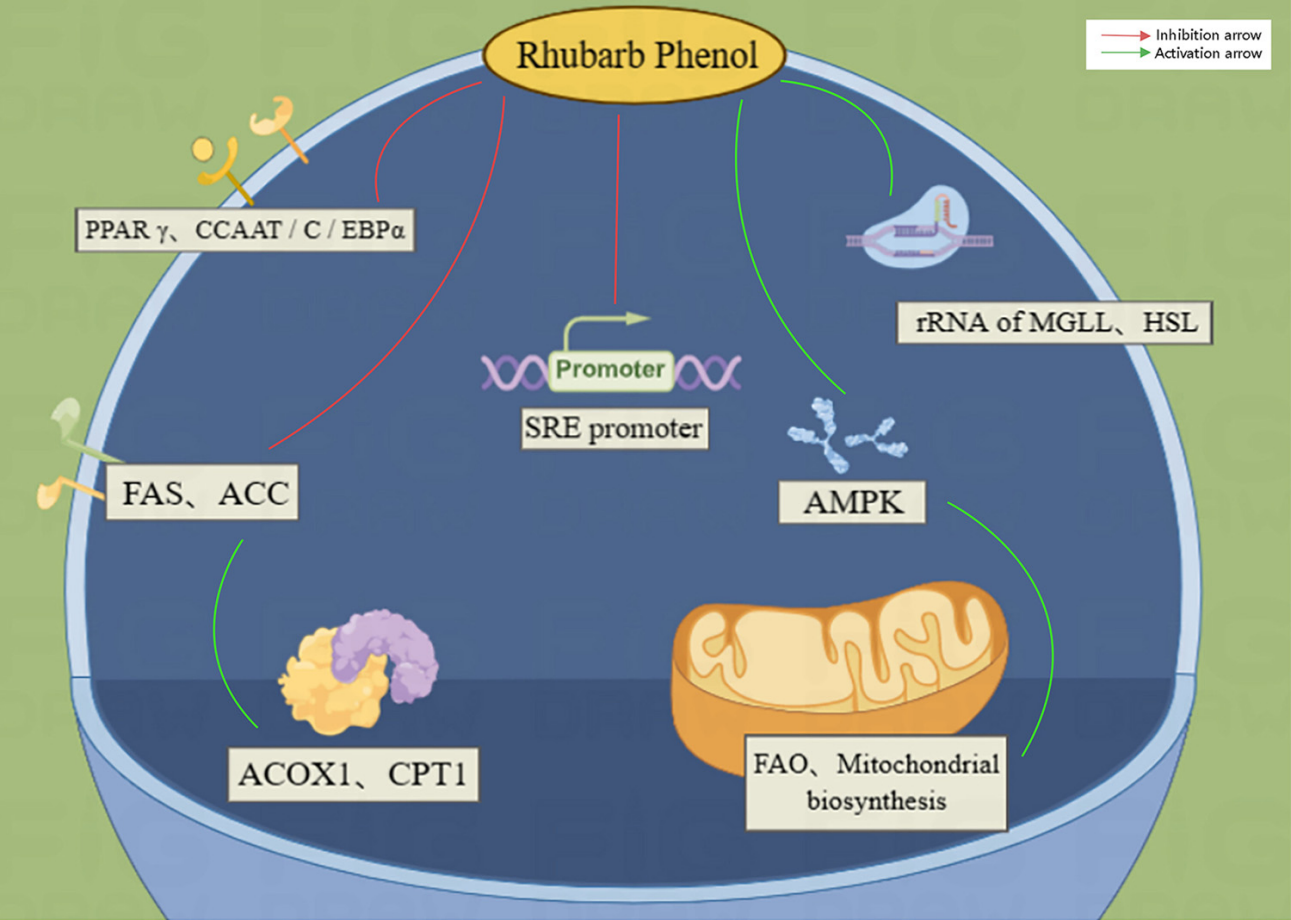
Diagram of the lipid-lowering mechanism of rhubarb phenol.

The hypolipidemic activity of rhubarb phenol is relatively weaker than that of emodin and rhubarb acid. It inhibits lipogenesis and promotes lipolysis, which is mainly realized through the regulation of FAS, ACC, the key enzymes MGLL and HSL, and oxidative genes (Figure 4). No serious adverse events were observed in studies on rhubarb phenol, suggesting its good safety profile.

### 3.4 Resveratrol

Resveratrol is a large group of astragalus compounds and an important component of rhubarb with anti-inflammatory, antioxidant, and anticancer properties [72]. Resveratrol can alter the gene expression profiles related to lipid metabolism [73]. For the first time, Zhang et al. proposed that resveratrol improves the lipid profile and reduces fat deposition *in vivo* in a porcine model, which may be mediated through fatty acid uptake, initial lipid synthesis, lipolysis, and FAO [74]. It has also been shown that the lipid-lowering mechanism of resveratrol mainly includes up-regulation of the expression of the cholesterol reverse transporter proteins PPARc and LXR α and some enzymes, modulation of the SIRT1-PPAR γ pathway and its downstream genes FAS and ACC, and increase in the ratio of apolipoproteins (APOs) A-I/ApoB [75,76]. Sahebkar et al. noted that in cell culture studies, resveratrol improved lipoprotein metabolism and reversed cholesterol transport while inhibiting foam cell formation [77]. In experimental models, resveratrol also exhibited antilipidemic activity by lowering LDL-C and TG levels and increasing HDL-C concentrations. Yuan et al. found that HFD-fed mice had dilated hepatocytes with significant lipid droplet accumulation, which was reduced by resveratrol treatment, further suggesting a lipid-modulating effect [78].

In terms of oxidative stress, Sebai et al. found that resveratrol reduces the pro-oxidant effects of the LPS-induced AR42J cell line through a Myd88-dependent signaling pathway [79] and through resveratrol intervention. TG levels can be reduced in T2DM patients [80], effectively reducing insulin resistance, lowering fasting blood glucose, and improving oxidative stress [81]. Its antioxidant effect was also demonstrated by the fact that the combination of resveratrol with antioxidant vitamins was more effective in protecting cells from oxidative stress than the antioxidants alone [82].

**Figure 5:**
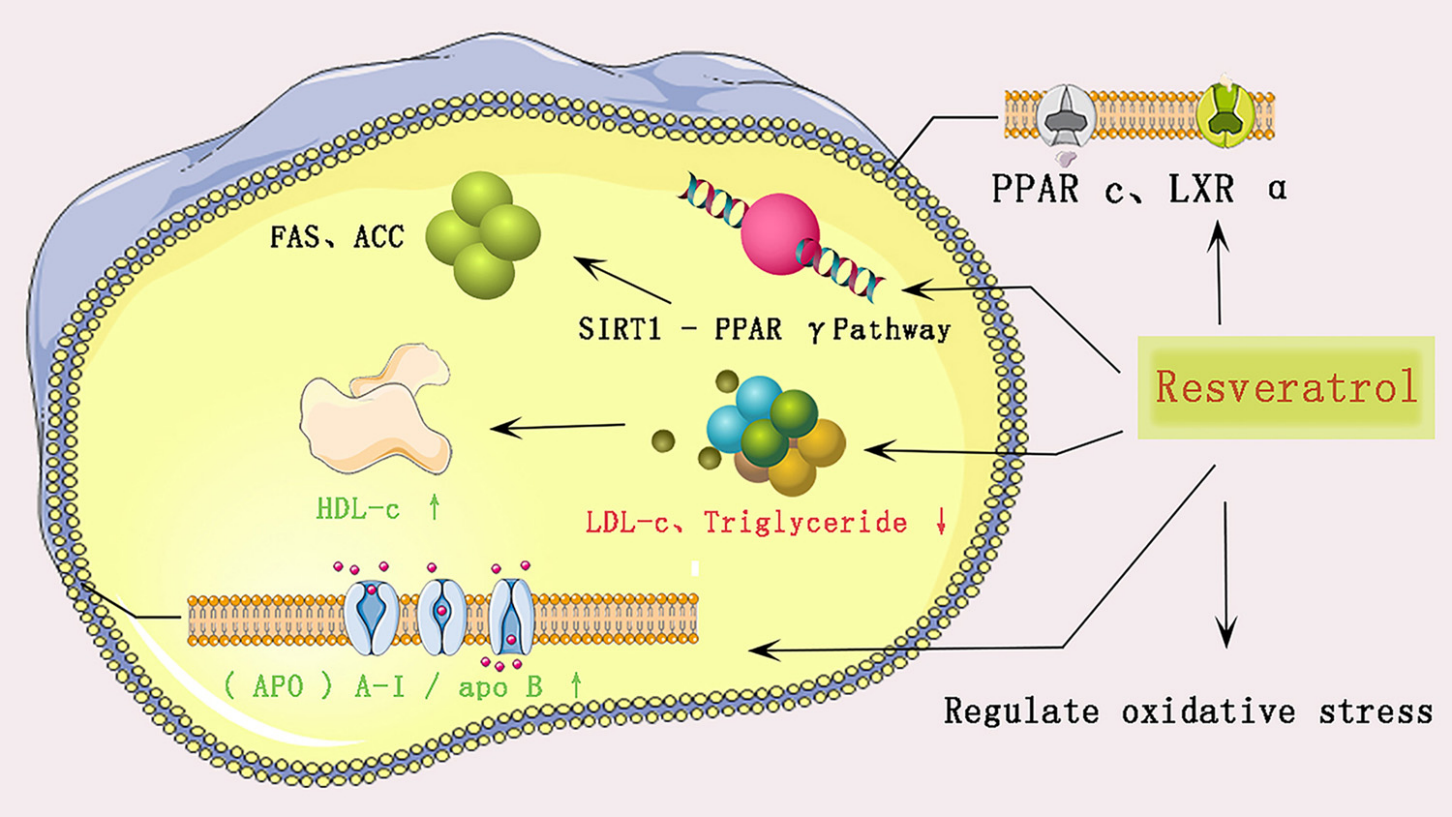
Diagram of the lipid-lowering mechanism of resveratrol.

Resveratrol, a common polyphenol found in astragalus, plays an important role in several chronic diseases, such as CVDs and obesity [79]. It achieves lipid lowering (Figure 5) and treatment of hyperlipidemia by modulating the lipid profile, SIRT1-PPAR γ pathway, lipoprotein metabolism, as well as promoting cholesterol transport and oxidative stress effects. However, similar to rhubarb acid, pharmacokinetic studies have shown that resveratrol has low solubility, rapid metabolism, and a short initial half-life [83]. To date, few studies have suggested solutions to address the low bioavailability and solubility of resveratrol; however, further definitive studies are needed to maximize its efficacy and increase its solubility.

### 3.5 Other components

In addition to rhodopsin, rhubarb acid, and rhubarb phenol, rhubarb-free anthraquinones, including rhubarb phenol methyl ether and aloe barbadensis rhubarb phenol, also exhibit hypolipidemic activity. Wang et al. found that the dichloromethane part of the ethanolic extract of rhubarb, which is mainly composed of rhubarb-free anthraquinones, has significant lipid-regulating effects that may enhance lipid metabolism, inhibit cholesterol synthesis [84], reduce peripheral LDLC and TC by inhibiting the PCSK9 gene, and promote intestinal cholesterol excretion by activating ABCG8 gene expression. Earth rhubarb glycosides and edible rhubarb sapogenins belong to the class rhubarb stilbenes. A study reported [36] that earth rhubarb glycosides significantly reduced the plasma TG, LDL, cholesterol, non-esterified free fatty acid, and insulin levels in KK/Ay type 2 diabetic mice. Jo et al. found that the consumption of rhubarb glycosides improved the pathological features of degenerative fatty liver in rats with hyperlipidemia induced by a high-cholesterol diet and significantly lowered blood lipid levels [85]. Additionally, rhubarb stem fiber has a hypolipidemic effect, which is thought to be due to its bile acid-binding capacity of rhubarb fiber, which in turn regulates cholesterol 7a-hydroxylase (cyp7a) activity [86].

Squalene cyclooxygenase (SE) (EC 1.14.99.7) is a nonmetallic flavoprotein monooxygenase that catalyzes the rate-limiting step in cholesterol biosynthesis [87]. Therefore, SE inhibitors become potential drugs for lowering cholesterol levels. Gallic acid derivatives of rhubarb are potent inhibitors of SE, a rate-limiting enzyme in cholesterol biosynthesis [88]. The other major constituents of rhubarb, senna A and dianthrone glucoside also showed favorable SE inhibitory effects [39]. In conclusion, the hypolipidemic effect of rhubarb has been clinically confirmed, and its chemical components and derivatives have shown either strong or weak hypolipidemic activity, which has far-reaching implications for the development of natural plant-based drugs against hyperlipidemia.

## 4 Toxic effects of rhubarb

The mechanisms underlying the toxic effects of rhubarb are not fully understood, and cells and animals in healthy or diseased states do not react to rhubarb in the same way. It has been confirmed that rhubarb has different degrees of toxicity in the liver, kidney, gastrointestinal tract, reproductive system, and blood system [89]. Studies have shown that the toxic effects of rhubarb are more pronounced in the liver and kidneys, and rhubarb affects the metabolism of endogenous substances such as mitochondria and bile acids through a series of adverse reactions, thus, causing liver damage [90,91]. Among these, anthraquinones and siderophores are closely related to the main toxic components of rhubarb [92], particularly because of substances such as emodin, aloe rhodopsin, and rhubarb acid. Animal experiments and clinical applications have confirmed significant bidirectional effects of rhubarb on hepatotoxicity and hepatoprotection. Dong et al. [93] examined the toxicity and target organs of rhubarb in rats using *in vivo* and *in vitro* experiments and found that emodin was the main toxic component. Based on the morphological changes in L-02 cells [94], rhodopsin (30 μM) causes significant apoptosis in a time-dependent manner. Additionally, emodin has the potential to interfere with GSH and fatty acid metabolism in human hepatocytes [95]. Wang et al. [96] studied the effect of total rhubarb extract in normal and pathological animals and found that rhubarb has hepatotoxicity in normal animals but has a protective effect against chronic liver injury caused by CCl4 damage. Particularly, cooked rhubarb after concoction has a stronger hepatoprotective effect with lower toxicity. Meanwhile, rhubarb benefits hepatocytes by scavenging free radicals; lowering the level of MDA, a key factor in liver inflammation; and increasing the total antioxidant capacity through oxidative stress, resulting in improved antioxidant damage, reduced lipid peroxidation, and stabilized cell membranes [92]. Rhubarb extract also has significant nephrotoxic and protective effects. The rhubarb extract emodin and rhubarb acid at a dose of 4.5 g/kg per day for 13 weeks induced a significant nephrotoxic effect in Sprague–Dawley rats. Rat renal tubular epithelial cells swell and degenerate [97], with significant cytotoxic effects. In a systematic evaluation, rhubarb showed positive effects in 1,322 patients with chronic kidney disease by alleviating uremic symptoms, lowering blood creatinine levels, improving hemoglobin levels, and regulating lipid metabolism disorders [98]. Another 6 months study showed that the critical dose of rhubarb extract required to induce nephropathy in rats was 10 g/kg body weight per day in raw doses, which recovered upon discontinuation of the drug [91]. No nephropathy was observed in normal rats after repeated administration of rhubarb extract at doses of 3 and 20 g/kg body weight per day (calculated using the crude amount) for 3 weeks [99].

It seems contradictory that rhubarb exhibits both toxic and protective effects on the liver and kidneys. Based on the literature, we found that the toxicity of rhubarb in the liver and kidneys was dose and time dependent. Therefore, we speculate that the reason for this contradiction lies in the dose and duration of administration. High-dose and long-term administration are more likely to induce hepatorenal toxicity, whereas low-dose and short-term administration may have protective effects. Additionally, owing to the bidirectional effect of rhubarb, it has been suggested that rhubarb may have hepatorenal protective potential in hyperlipidemia, fatty liver, and chronic renal failure; however, the specific mechanism requires further study.

## 5 Discussion

Hyperlipidemia is a serious threat to human health, and long-term hyperlipidemia can lead to atherosclerosis, coronary heart disease, peripheral vascular disease, ischemic cerebrovascular disease, pancreatitis, and other chronic diseases. Disorders in lipid metabolism can significantly affect the occurrence and development of metabolic diseases. As a traditional Chinese medicine, rhubarb, with its precise lipid-lowering efficacy, provides a new direction for the treatment of hyperlipidemia. Based on the large body of literature on the pharmacological components of rhubarb, this study summarizes the lipid-lowering molecular mechanisms of some of its chemical components, providing theoretical support for the clinical application of rhubarb in the treatment of hyperlipidemia. However, most studies have focused on chemical mechanism exploration and preclinical studies, and there is a lack of strong clinical data to confirm the therapeutic effects of rhubarb on hyperlipidemia. Although approximately 200 compounds of rhubarb have been identified in phytochemistry, they are mainly emodin, rhubarb acid, rhubarb phenol, and other important chemical constituents that exert a hypolipidemic effect. Therefore, the lipid-lowering effect of rhubarb is closely related to the contents of these important chemical components. Additionally, we found that the toxic effects of rhubarb are influenced by the content of these chemical components. Particularly, the bidirectional nature of its toxic and protective effects suggests the dose–effect and toxicity–effect relationships of rhubarb in the therapeutic process. The synergistic effect of different substances is a promising research trend, for example, whether better efficacy can be obtained by combining the main lipid-lowering components of rhubarb extract with other existing natural or synthetic drugs.

Despite reviewing the hypolipidemic effects of rhubarb in the present study, some limitations remain. First, the hypolipidemic activity of the chemical constituents of rhubarb has been described in several studies. However, cellular and animal model studies of rhubarb in the treatment of hyperlipidemia are limited, and there is a lack of experimental data from large samples. Second, rhubarb can be used as a lipid-lowering drug; however, there is a lack of comparative toxicity data with existing lipid-lowering drugs, and it is not known whether it can replace commonly used clinical hyperlipidemia drugs. Although rhubarb has a better lipid-lowering effect, there are differences in the specific composition of rhubarb from different regions and varieties, and further investigation is needed to determine whether this affects the lipid-lowering effect of rhubarb.

As a potential candidate for the treatment of hyperlipidemia, we still need to address the following questions before using rhubarb for clinical use: (1) The use of rhubarb as a Chinese herbal medicine will inevitably be disturbed by external factors, such as the boiling method, time, and container, and whether this will affect the lipid-lowering activity and solubility of important components, such as rhodopsin and rhubarb acid. (2) To clarify the reasonable dose and administration time of rhubarb for the treatment of hyperlipidemia, its safety should be improved. (3) The low bioextractability of the main lipid-lowering components of rhubarb is also a considerable challenge in improving its preparation process. Additionally, the combination of rhubarb with nanomaterials or novel drug delivery systems can reduce its toxicity and improve its bioavailability.

## 6 Conclusion

In this article, we present a complete review of the main active components and mechanisms of action of rhubarb in lowering lipid levels. Our results showed that the main components of rhubarb involved in lipid metabolism were anthraquinones and stilbene compounds, including emodin, rhubarb acid, rhubarb phenol, and resveratrol. Its specific mechanisms of action are mainly related to the reduction of lipogenesis, stimulation of lipolysis, and inhibition of gene expression, especially in 3T3-L1 adipocytes, TNF-α inflammatory mediators, PPAR α, C/EBP α, Myd88, and MAPK signaling pathways, as well as the lipid metabolism of enzymes such as ATGL, HSL, MGLL, and other transcription factors. Therefore, the multi-component and multi-target lipid-lowering effects of rhubarb make it a potential natural drug for the treatment of hyperlipidemia.

## Abbreviations

APOs: apolipoproteins
CVD: cardiovascular disease
FAO: fatty acid oxidation
GSH: glutathione
HDL-C: high-density lipoprotein cholesterol
HFD: high-fat diet
LDL-C: low-density lipoprotein cholesterol
PPAR: peroxisome proliferator-activated receptor
SE: squalene cyclooxygenase
TC: total cholesterol
TG: triglyceride
